# The complete chloroplast genome sequence of *Clivia miniata* var. *citrina*

**DOI:** 10.1080/23802359.2021.1948363

**Published:** 2021-11-28

**Authors:** Xing-Hua Zhao, Ling Yue, Xiu-Li Feng, Dan Li, Hai-Hong Wu

**Affiliations:** Institute of Floriculture, Liaoning Academy of Agricultural Sciences, Shenyang, China

**Keywords:** *Clivia miniata* var*. citrina*, chloroplast genome, phylogenetic analysis

## Abstract

The complete chloroplast genome of *Clivia miniata* var*. citrina* was assembled and subjected to phylogenetic analysis in this study. The complete chloroplast genome of *C. miniata* var*. citrina* was 158,112 bp in length, containing a large single-copy region (LSC, 86,202 bp), a small single-copy region (SSC, 18,334 bp), and two inverted repeat regions (IRs, 26,788 bp). The GC content was 37.97%. A total of 130 genes were annotated, including 86 protein-coding genes, 36 tRNA and 8 rRNA genes. Phylogenetic analysis showed that *C. miniata* var*. citrina* was the most related with *C. miniata* and they formed a monophyletic group that was sister to the clade of *Hippeastrum, Leucojum, Narcissus* and *Lysoris*.

*Clivia miniata* var*. citrina* S. Watson, an evergreen monocot with rhizomes, is belonged to the family Amaryllidaceae. It is widely used as a cultivated ornamental perennial herb and additionally has high medicinal values. In this study, the complete chloroplast genome sequence of *C. miniata* var*. citrina* was assembled and subjected to phylogenetic analysis, to reveal its evolution relationships and provide genome resources for breeding of horticultural varieties of *Clivia*.

The plant material of *C. miniata* var*. citrina* was sampled from Liaoning Academy of Agricultural Sciences (N41°48′33″, E123°34′53″), Shenyang, China. The specimen was stored with the archival number HHJZL01 at the Institute of Floriculture of Liaoning Academy of Agricultural Sciences. The total genomic DNA was extracted using the modified CTAB method (Doyle and Doyle 1987). A genomic shotgun library with an insertion size of 400 bp was constructed using TruSeq DNA Sample Prep Kit and subsequently sequenced using the 2 × 150 bp paired-end mode on the Illumina NovaSeq platform. The complete chloroplast genome was assembled by using GetOrganelle v1.6.2e (Jin et al. 2020) with the built-in reference sequences, and then annotated with the OGAP pipeline (https://github.com/zhangrengang/OGAP). The complete chloroplast genome sequence of *C. miniata* var*. citrina* had been deposited in the GenBank (accession number MW561118).

The complete chloroplast genome of *C. miniata* var*. citrina* was 158,112 bp in length, with a total GC content of 37.97%. It consisted of a large single-copy region (LSC, 86,202 bp), a small single-copy region (SSC, 18,334 bp), and two inverted repeat regions (IRs, 26,788 bp). The chloroplast genome contained a total of 130 complete genes, including 86 protein-coding genes, 36 tRNA and 8 rRNA genes. There were 20 genes duplicated in the IR regions, including 7 protein-coding genes, 9 tRNA and 4 rRNA genes.

To evaluate the phylogenetic relationship of *C. miniata* var*. citrina* within Amaryllidaceae, twelve representative species of the family Amaryllidaceae were selected, with *Lilium brownii* (Liliaceae) as an outgroup. Complete chloroplast genome sequences were aligned using MAFFT v7.471 (Standley and Katoh 2013) and the multiple alignment was trimmed using trimAl v1.2 (Capella-Gutierrez et al. 2009). The maximum likelihood phylogenetic tree was reconstructed by using IQ-TREE v1.6.5 (Nguyen et al. 2015), with the best-fit model of TVM + F+R3 and 1000 bootstrap replicates. The result showed that *C. miniata* var*. citrina* was the most related with *C. miniata* and they formed a monophyletic group that was sister to the clade of *Hippeastrum*, *Leucojum*, *Narcissus* and *Lycoris* (Figure 1). The degree of differentiation between *C. miniata* var*. citrina* and *C. miniata* was 0.0013%.

**Figure 1.**
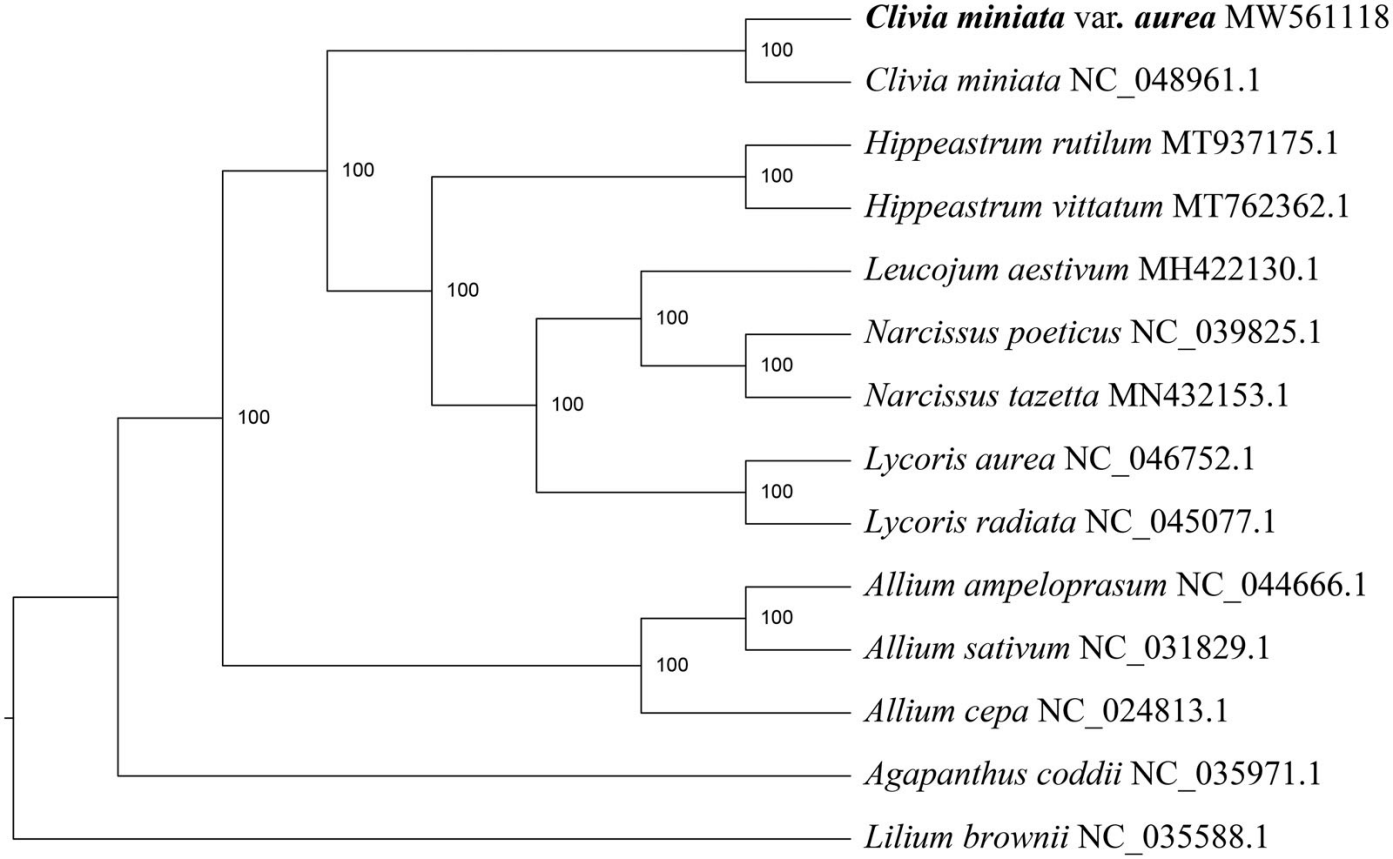
Maximum-likelihood phylogenetic tree based on chloroplast whole-genome sequences showing relationships of *Clivia miniata* var*. citrina* with representative members of the family Amaryllidaceae. *Lilium brownii* (Liliaceae) was served as the outgroup. Numbers at nodes represent bootstrap percentage values from 1000 replicates.

## Data Availability

The data that support the analyses and results of this study are openly available in Genbank with accession MW561118 (https://www.ncbi.nlm.nih.gov/nuccore/MW561118). Sequencing reads was deposited in Sequence Read Archive (SRA) with BioProject accession PRJNA702545 (https://www.ncbi.nlm.nih.gov/bioproject/PRJNA702545).
